# Supplementary material for: Investigating the Effectiveness of a Workplace Musculoskeletal Disorders Management Program
Source: Healthcare (Basel). 2024 Sep 10;12(18):1815. doi: 10.3390/healthcare12181815 (PMC11431167; doi:10.3390/healthcare12181815)
Supplement: Supplementary file 1 [file healthcare-12-01815-s001.zip › healthcare-3158764-supplementary file 1 - Table and Figure.pdf]

Supplementary material Tables and Figures

Table S1. Work Productivity and activity impairment questionnaire (WPAI). Comparison of the variables only within the early intervention group at the three time points.

Early Intervention Group	Baseline/ Mean	Early evaluation/ Mean	Late evaluation/ Mean	P-value between baseline and early evaluation	P-value between baseline and late evaluation	P-value between early evaluation and late evaluation
% of working time lost due to health	0.01	0.04	0.01	0.209	0.468	0.2
% of limited capacity during work due to health	0.11	0.11	0.07	0.456	0.299	**0.045**
% of overall limited work capacity due to health	0.11	0.12	0.08	0.59	0.191	**0.048**
% of limited activity due to health	0.16	0.13	0.10	0.11	**0.025**	0.096

[a] t-test for repeated measures.

Table S2. Work Productivity and activity impairment questionnaire (WPAI). Comparison of the variables only within the late intervention group at the three time points.

Late Intervention Group	Baseline 1/ Mean	Baseline 2/ Mean	Late evaluation/ Mean	P-value between baseline 1 and baseline 2 evaluation	P-value between baseline 1 and late evaluation	P-value between baseline 2 and late evaluation
% of working time lost	0.01	0	0.01	0.635	0.391	0.196

Late Intervention Group	Baseline 1/ Mean	Baseline 2/ Mean	Late evaluation/ Mean	P-value between baseline 1 and baseline 2 evaluation	P-value between baseline 1 and late evaluation	P-value between baseline 2 and late evaluation
due to health						
% of limited capacity during work due to health	0.09	0.1	0.09	0.908	0.672	0.502
% of overall limited work capacity due to health	0.1	0.1	0.1	0.948	0.906	0.738
% of limited activity due to health	0.13	0.12	0.1	0.723	0.154	0.297

[a] **t-test for repeated measures.**

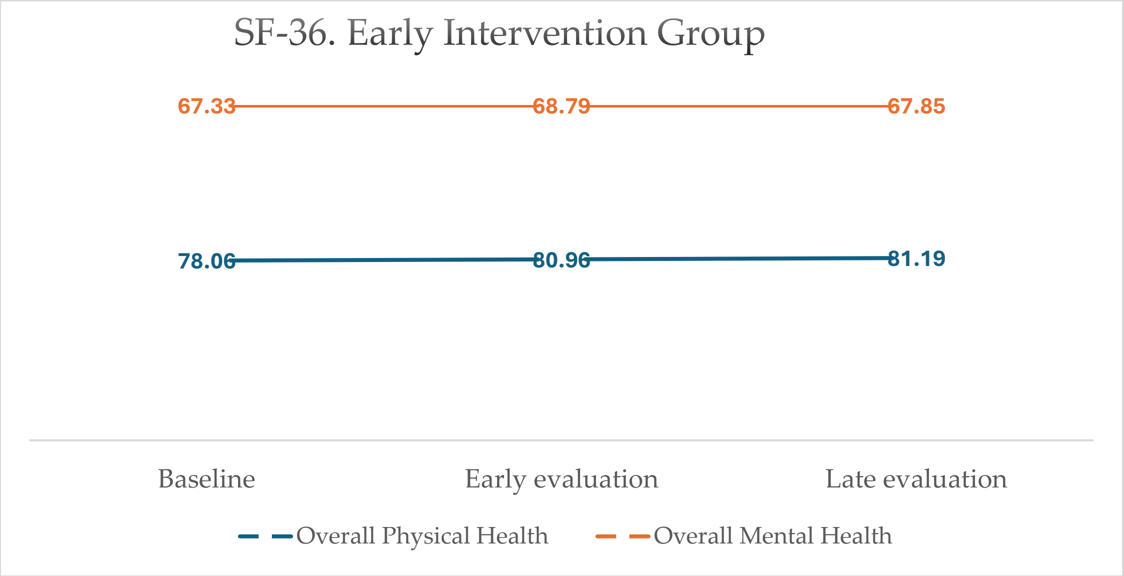

Figure S1. SF-36 questionnaire. Comparison of the variables only within the early intervention group at the three time points

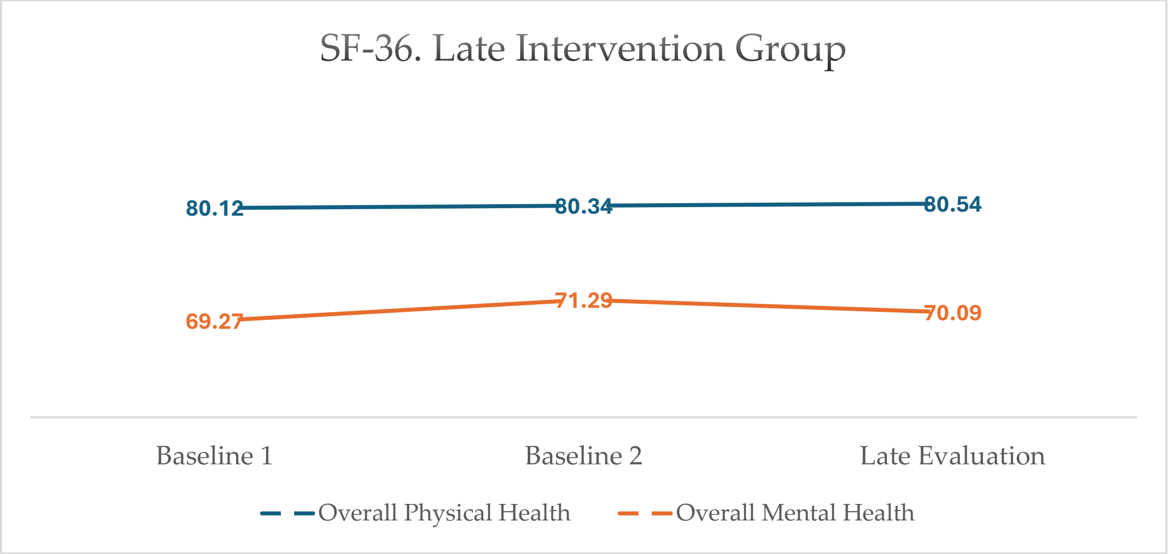

Figure S2. SF-36 questionnaire. Comparison of the variables only within the late intervention group at the three time points

Table S3. Standardised Nordic questionnaire. Comparison of the variables only within the early intervention group at the three time points.

	Baseline	Early evaluation	Late evaluation	P-value between baseline and early evaluation	P-value between baseline and late evaluation	P-value between early evaluation and late evaluation
	Percentage	Percentage	Percentage			
**Neck**	47	49	47	0.118	1	0.659
**Neck last 12 months**	16	12	4	0.81	**0.032**	**0.044**
**Neck last 7 days**	41	33	18	0.184	**0.004**	**0.004**
**Shoulders**	76	73	53	**0.015**	0.204	0.058
**Shoulders last 12 months**	20	14	12	0.059	0.209	0.569
**Shoulders last 7 days**	22	27	16	0.338	0.411	**0.013**
**Elbows**	18	14	14	0.071	0.42	1
**Elbows last 12 months**	6	4	2	0.657	0.322	0.322
**Elbows last 7 days**	4	4	4	0.368	1	0.159
**Wrists**	41	47	22	0.087	0.159	**0.014**
**Wrists last 12 months**	12	12	6	0.083	0.261	0.083
**Wrists last 7 days**	10	20	10	1	1	**0.024**
**Upper back**	24	14	14	0.09	0.133	1
**Upper back last 12 months**	6	8	0	0.741	0.083	**0.044**
**Upper back last 7 days**	18	10	2	0.619	**0.004**	**0.044**
**Lower back**	35	29	22	0.1	0.07	**0.044**
**Lower back last 12 months**	22	14	4	**0.049**	**0.011**	**0.024**
**Lower back last 7 days**	25	14	10	**0.041**	**0.031**	0.159
**One or both Hips/Thighs**	22	16	14	0.059	0.159	0.322
**Hips/Thighs last 12 months**	10	2	2	0.058	**0.044**	0.322
**Hips/Thighs last 7 days**	18	10	6	0.132	**0.032**	0.159
**One or both knees**	27	22	20	**0.038**	0.159	0.322
**Knees last 12 months**	12	10	4	0.259	0.103	0.083

**Knees last 7 days**	16	10	2	0.096	**0.018**	**0.044**
**One or both ankles/feet**	14	12	8	0.25	0.261	0.159
**Ankles/feet last 12 months**	6	2	0	0.058	0.083	0.322
**Ankles/feet last 7 days**	10	4	0	0.158	**0.024**	0.159

[a] McNemar test.

Table S4. Standardised Nordic questionnaire. Comparison of the variables only within the late intervention group at the three time points.

	Baseline 1	Baseline 2	Late evaluation	P-value between baseline 1 and baseline 2 evaluation	P-value between baseline 1 and late evaluation	P-value between baseline 2 and late evaluation
	Percentage	Percentage	Percentage			
**Neck**	60	63	48	0.81	0.49	**0.01**
**Neck last 12 months**	21	23	15	1	0.096	0.083
**Neck last 7 days**	46	45	29	0.657	**0.005**	**0.019**
**Shoulders**	54	46	31	0.138	**0.017**	0.091
**Shoulders last 12 months**	21	26	7	0.202	**0.004**	**<0.001**
**Shoulders last 7 days**	41	41	25	1	**0.011**	**0.019**
**Elbows**	13	15	12	0.426	1	0.686
**Elbows last 12 months**	4	5	4	0.566	1	0.658
**Elbows last 7 days**	6	5	6	0.708	1	0.708
**Wrists**	45	51	21	0.449	**0.029**	**0.005**
**Wrists last 12 months**	20	20	4	0.81	0	**0.002**
**Wrists last 7 days**	21	21	14	0.82	0.159	0.203
**Upper back**	21	30	15	0.181	0.254	**0.01**
**Upper back last 12 months**	11	10	5	0.741	0.096	0.251
**Upper back last 7 days**	16	18	9	0.798	0.109	0.052
**Lower back**	40	30	24	0.145	**0.008**	0.3
**Lower back last 12 months**	25	16	11	0.052	**0.002**	0.32

**Lower back last 7 days**	26	23	14	0.64	**0.024**	0.145
**One or both Hips/Thighs**	14	15	11	0.596	0.53	0.369
**Hips/Thighs last 12 months**	9	10	4	0.741	0.103	0.096
**Hips/Thighs last 7 days**	5	10	9	0.158	0.259	0.783
**One or both knees**	21	26	26	0.64	0.208	1
**Knees last 12 months**	10	11	6	0.741	0.181	0.208
**Knees last 7 days**	15	18	19	0.596	0.32	0.82
**One or both ankles/feet**	10	15	7	0.198	0.418	0.135
**Ankles/feet last 12 months**	5	6	4	0.657	0.658	0.483
**Ankles/feet last 7 days**	4	4	4	1	1	1

[a]McNemar test.

Table S5. Post intervention MSD pain (answer yes) by sex in both groups.

	Early intervention group			Late intervention group		
	**Male**	**Female**	**p-value**	**Male**	**Female**	**p-value**
**Neck last 12 months**	0	2/3.9%	0.5	4/5%	8/10%	0.367
**Neck last 7 days**	3/5.9%	6/11.76%	0.714	6/7.5%	17/7.5%	**0.027**
**Shoulders last 12 months**	2/3.9%	4/7.8%	0.688	1/1.25%	5/6.25%	0.209
**Shoulders last 7 days**	2/3.9%	6/11.76%	0.44	7/7.85%	13/16.25%	0.305
**Elbows last 12 months**	0	1/1.96%	0.431	1/1.25%	2/2.5%	1
**Elbows last 7 days**	1/1.96%	1/1.96%	1	1/1.25%	4/5%	0.366
**Wrists last 12 months**	1/1.96%	2/3.9%	1	1/1.25%	2/2.5%	1
**Wrists last 7 days**	1/1.96%	4/7.8%	0.375	3/3.75%	8/10%	0.208
**Upper back last 12 months**	0	0	0	2/2.5%	2/2.5%	1
**Upper back last 7 days**	0	1/1.96%	1	0	1/1.25%	1
**Lower back last 12 months**	0	2/3.9%	0.5	10/12.5%	9/11.25%	0.603
**Lower back last 7 days**	1/1.96%	4/7.8%	0.375	6/7.5%	4/5%	0.501
**Hips/Thighs last 12 months**	1/1.96%	0	0.431	2/2.5%	1/1.25%	0.597
**Hips/Thighs last 7 days**	0	3/5.9%	0.249	1/1.25%	6/7.5%	0.116
**Knees last 12 months**	0	2/3.9%	0.5	3/3.75%	2/2.5%	0.658
**Knees last 7 days**	0	1/1.96%	1	7/8.75%	8/10%	1
**Ankles/feet last 12 months**	0	0	0	2/2.5%	1/1.25%	0.593
**Ankles/feet last 7 days**	0	0	0	1/1.25%	2/2.5%	1
